# Associations Between Depression Symptoms, Psychological Intervention and Perinatal Complications

**DOI:** 10.1007/s10880-019-09632-4

**Published:** 2019-05-29

**Authors:** Emőke Adrienn Hompoth, Zoltán Pető, Veronika Fűrészné Balogh, Annamária Töreki

**Affiliations:** 1 Department of Personality, Clinical and Health Psychology, University of Szeged, Egyetem Street 2, Szeged, 6722 Hungary; 2 Department of Emergency Medicine, University of Szeged, Szeged, Hungary; 3 Department of Obstetrics and Gynecology, University of Szeged, Szeged, Hungary

**Keywords:** Antenatal and postpartum depression screening, Antenatal care, Postnatal care, Psychological intervention, Obstetric complications

## Abstract

Antenatal and postpartum depression has been associated with maternal, child and family-unit complications. Our aim was to assess the impact of a depression screening and intervention program on perinatal complications. This study included 2042 women. They were screened on the Edinburgh Postnatal Depression Scale (EPDS), three times during pregnancy and once after childbirth. If their EPDS score was above the cut-off score, psychological intervention was offered. Significant relationships were found between depression scores and perinatal complications, such as protracted cervical dilation, protracted descent, preeclampsia, intrauterine growth restriction, low birthweight and cesarean section. Depression scores were higher in the intervention group, compared to the non-intervention group, but decreased after the consultations. The cesarean section rate was significantly lower in the consultation group. A rapid screening process can provide an adequate tool to identify women who are more likely to have such complications due to depression.

## Introduction

Pregnancy and the postpartum period can be very demanding and women need well-functioning cognitive skills—such as time-management skills, a well-functioning memory and decision-making skills—to be able to manage all the challenges that motherhood brings. If these cognitive functions are altered due to postpartum depression, the infant can be adversely affected; therefore, the condition of the mother is considered to be an important healthcare issue (Logsdon, Wisner, & Pinto-Foltz, 2006).

Untreated depression was shown to be a risk factor for unfavorable pregnancy outcomes (Marcus, 2009) such as increased substance use, underutilization of antenatal care and inadequate weight gain (Marcus & Flynn, 2008). Depression is also associated with preterm birth (Jesse, Seaver, & Wallace, 2003), intrauterine growth restriction (Grote, Bridge, Gavin, Melville, Iyengar, & Katon, 2010), low birthweight (Rahman, Bunn, Lovel, & Creed, 2007; Hompoth, Töreki, B. Fűrész, & Németh, 2017) and a higher rate of cesarean section (Chung, Lau, Yip, Chiu, & Lee, 2001).

Known risk factors for developing depression in the antenatal phase are low self-esteem, antenatal anxiety, low social-support and negative cognitive style, but major life events can lead to depression symptoms, as well (Leigh & Milgrom, 2008). Postpartum risk factors are antenatal depression and anxiety, childcare stress, low social-support, an unsatisfactory marital relationship, unplanned or unwanted pregnancy (Beck, 2001) and cesarean section (Kovácsné Török, 2009). To minimize the potentially harmful effects of the mother’s depression on her infant, preventive measures and early identification and provision of treatment are very important (Beck, 2001). Effective management of the depression symptoms can be difficult, since women rarely seek treatment (Logsdon et al., 2006). This might be partly because they often think that these depression symptoms are due to pregnancy-related changes (O’Mahen, Flynn, Chermack, & Marcus, 2009). In addition, women are often afraid of the social stigma associated with postpartum depression (Beck, 2001). According to Flynn, O’Mahen, Massey and Marcus (2006), when pregnant women are informed about depression symptoms by their physicians, they are more likely to seek treatment before their next antenatal follow-up. Those women who had a serious fear of childbirth and attended psychological intervention (psychoeducation and relaxation) were significantly more likely to choose vaginal birth instead of elective cesarean section (Saisto, Toivanen, Salmela-Aro, & Halmesmäki, 2006). In Hungary, pregnant women do not have the opportunity to choose between vaginal birth and cesarean section. We wanted to investigate whether psychological intervention is connected to lower rates of cesarean section, because we found no reference to this topic in the literature.

The depression screening program in the antenatal and postpartum period in X started on April 2011 and is still fully operational. In our work, the primary aims were to assess the prevalence of depression symptoms during the antenatal and postpartum periods and to determine whether the screening tool scores are related to antenatal and obstetric complications. We also wanted to know how the psychological consultations affected depression scores and obstetric outcomes within the study group. Our hypotheses (H) were as follows:

- *H1* women in the complication groups (specified in the statistical analysis part of this paper) will have higher depression scores compared to women in the control group
- *H2* the depression scores of women who had a cesarean section will be higher in the antenatal and postpartum phase compared to women who had a vaginal delivery
- *H3* the depression scores will be lower after the interventions compared to the before-intervention state
- *H4* women in the intervention group will have a lower complication rate compared to women in the non-intervention group
- *H5* The cesarean section rate of women who attended the intervention during their pregnancy will be lower compared to women in the non-intervention group.

## Materials and Methods

### Ethical Approval

The study was approved by the Clinical Research Ethics Committee of the University of Szeged (100/2010) and was carried out according to the Declaration of Helsinki and the Oviedo Convention.

### Sample

Since 2011, 4593 women took part in the depression screening program. The only exclusion criterion was lack of fluency in Hungarian. In the last 3 years, 2118 women’s obstetric data were collected from our patient-record system. We excluded 26 women with twins and 50 women with a procured or spontaneous abortion or stillbirth from the study. Thus, 2042 women were included in the study sample.

### Screening Tool

The Edinburgh Postnatal Depression Scale (EPDS)—created by Cox, Holden and Sagovsky (1987) and translated into the Hungarian language and validated by Töreki et al. (2013, 2014)—was used as the screening tool. This is a short, ten-item questionnaire that measures the mood status of the previous week. For each question, women can choose from four answers, which are scored from 0 to 3 points. The 10th question of which measures suicidal tendency: women have to indicate how often they think about harming themselves. We used the validated depression cut-off scores of 6/7 in the antenatal period, and 7/8 in the postpartum phase. This means that until 6 points in the antenatal period, and 7 points in the postpartum phase, the result was in the normal range, but above these figures depression was probable.

It is important to emphasize that the EPDS is a screening tool, not a diagnostic one. We use the terms “depression” or “depressed” only for better understanding; we do not want to imply that we diagnosed these women as having a clinical disorder.

### Screening Procedure and Study Design

Perinatal nurses play a major role in the screening process, as they meet pregnant women regularly in every trimester, and during the postpartum period, as part of pregnancy care. At the first pregnancy care meeting (usually in the first trimester), the perinatal nurse explained the essence and aims of the screening, then she provided women with the written informed consent form, the demographic data sheet and, finally, the EPDS questionnaire. Women filled-out the EPDS three more times, once during the second trimester, again during the third trimester, then once more during the postpartum period (between weeks 4 and 6).

Perinatal nurses sent the questionnaires to the Department of Obstetrics and Gynecology psychologist, who registered the results into the patient-record system. As part of the screening program, psychological consultation was offered and provided, both in the antenatal and the postpartum period, to those women who scored above the cut-off value.

Because each woman in our sample filled out the EPDS questionnaire several times, our study design is Repeated Measures Within-Subject design.

### Intervention

From the 2118 women whose obstetric data were collected, 188 women asked for psychological consultation as part of the screening program. From these 188, we excluded 38 women with twins and women who had a procured or spontaneous abortion or stillbirth. The remaining 150 women constituted the intervention group. The remaining 1892 women made up the non-intervention group.

During the psychological intervention sessions, two health psychologists explored the features of symptoms more deeply, including onset, changes in intensity and their impact on women’s everyday lives. The level of support provided by her family, friends and surroundings was assessed, as well. The content and duration of sessions were not strictly predetermined, and the problems most bothering the individual were focused upon. The number of sessions was also adjusted to each woman’s needs. The intervention style was person-to-person and each session lasted a nominal 45 min. The main goals of the interventions were to support women in maintaining healthy function and to help them to cope with symptoms.

The methods used by the psychologist were the following: supporting and reinforcement, psychoeducation, relaxation techniques, crisis-intervention, enhancing coping skills and sense of control, reducing anxiety, hopelessness and depression symptoms, providing assistance in coping with various losses, relationship counselling and cognitive-behavioral elements. When psychiatric intervention appeared to, perhaps, be required, women were directed to a medical care specialist in the Department of Psychiatry at University of Szeged.

### Statistical Analysis

We used IBM SPSS Statistics, version 22, for statistical analysis. We set the significance level to 0.05. We created groups based on the EPDS scores, the complications, mode-of-birth and attendance at the interventions.

Using the women’s obstetric data, we created seven complication groups: (1) preterm birth (under 36 weeks), (2) protracted cervical dilation, (3) protracted descent, (4) preeclampsia, (5) gestational diabetes mellitus (GDM), (6) intrauterine growth restriction (IUGR) and (7) low birthweight (under 2500 g). The control group was made up of women who did not have any of these complications. Mode-of-birth was divided into two groups: cesarean section (with emergency and elective cesarean section) and vaginal birth. Women who attended the interventions constituted the intervention group and everyone else was in the non-intervention group.

To enhance the power of the analyses, we supplemented the missing data of the EPDS scores: we calculated the median value of the scores of the four measuring occasions separately for the following groups: the seven complication groups and (8) women who had two or more of the above-mentioned complications and (9) women who had none of the above-mentioned complications. We used this method because previous studies have found connections between depression and most of these complications; thus, calculating only one median value per measuring occasion could have distorted the results. In all, we supplemented 182 scores in the first trimester, 404 and 646 scores in the second and third trimesters, respectively, and 903 scores in the postpartum period.

We used non-parametric tests in the statistical analyses because these methods do not require a normal distribution. These tests transform the data (the EPDS scores) in their computations, and their results are mean ranks (instead of EPDS score means). The results are a little harder to interpret: they cannot be directly compared to the EPDS scores, but these methods are more reliable when the variables are not normally distributed, although higher mean ranks refer to more depression symptoms, just like higher EPDS scores.

We explored pathological rates using Frequencies analysis on depression and non-depression groups in each trimester. Friedman’s test was used to analyze whether EPDS mean ranks change over time, as pregnancy proceeds and after the child is born. Mann–Whitney *U* tests with Bonferroni Corrections were used to analyze whether EPDS mean ranks were connected to perinatal complications. A Mann–Whitney *U* test was used to determine whether there was any connection between the EPDS mean ranks and mode-of-birth.

A Kruskal–Wallis test with pairwise comparisons was used to analyze whether there was a difference in the EPDS mean ranks between women who started the intervention in different trimesters or did not attend at all. Pearson’s *χ*^2^ tests were used to determine whether there were any connections between intervention and perinatal complications and mode-of-birth. Non-supplemented EPDS scores and a Wilcoxon Signed-Rank test were used to explore whether the EPDS mean ranks changed after the consultations compared to the before-intervention state.

## Results

### Sample Characteristics

The responding women’s ages were 15–44 years, and the mean age of the sample was 30.43 years (SD = 4.859). Some 1527 women (82.4%) were in a relationship, engaged or married; 1048 women (56.3%) did not have any children yet and 218 women (11.8%) had not planned their pregnancy.

The EPDS questionnaires were obtained around week 10.59, 22.44 and 34.43 during pregnancy, and week 4.81 after childbirth. According to the scores, 16.8% of the respondents were depressed in the first trimester, 12.2% and 10.5% in the second and third trimester, and 7.7% in the postpartum phase.

We analyzed the demographic characteristics of the intervention and non-intervention group, too. In the non-intervention group, the responding women’s ages ranged between 15 and 44 (mean = 30.45 SD = 4.182). Some 1428 women (83%) were in a relationship, engaged or married; 963 women (55.8%) did not have any children yet, and 184 women (10.8%) had not planned their pregnancy. In the intervention group, the responding women’s ages ranged between 16 and 43 (mean = 30.09 SD = 5.454). Some 99 women (74.4%) were in a relationship, engaged or married; 85 women (63.4%) did not have any children yet, and 34 women (25.4%) had not planned their pregnancy. As we can see, women in the intervention group were less likely to be in a relationship, more likely to be having their first child, and almost two-and-a-half times more likely to be having an unplanned pregnancy.

### Intervention and EPDS Scores, Participation Rate

According to the EPDS scores, 1561 (76.44%) women did not need intervention, but 37 of them came anyway, because they felt they needed to. In all, 481 (23.56%) women were offered consultation because their EPDS scores were over the cut-off value, but only 113 (23.5%) of them attended.

The number of sessions varied from one to twenty, the mean being 3.4 (SD = 3.9). The problems that were the focus of the interventions also varied: stress, low mood, panic symptoms, anxiety and obsessive thoughts, relationship difficulties and conflicts with family, but women talked about feelings of being a bad mother, fear of birth, previous abortion or stillbirth and posttraumatic stress symptoms from previous cesarean section, too. Unplanned pregnancy and uncertainty about wanting the child were also common topics.

The Wilcoxon Signed-Rank test showed that the EPDS mean rank significantly decreased after the consultations compared to the before-intervention state, from 38.75 to 25.57, *p* < .001, *N* = 78.

**Fig. 1:**
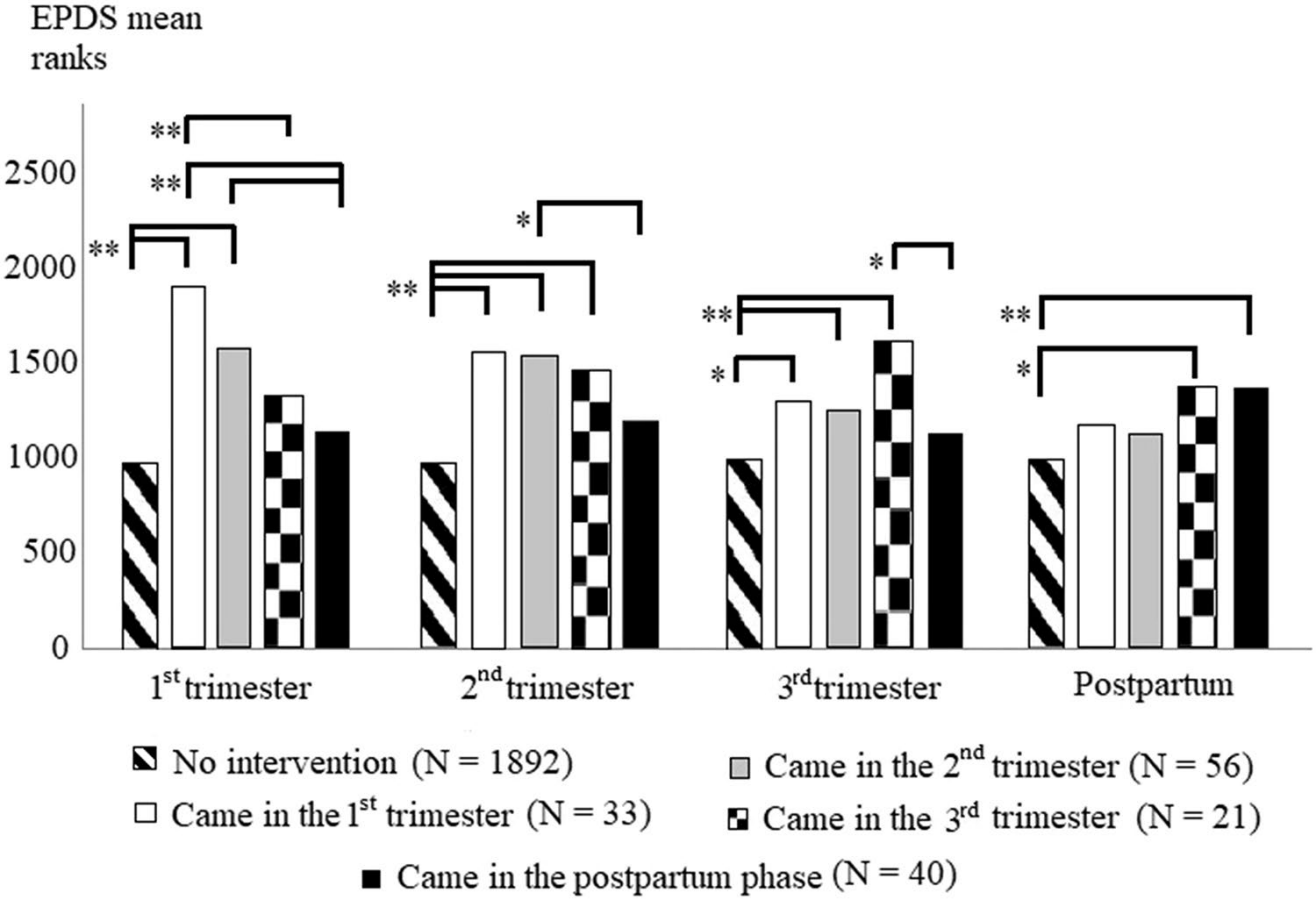
Adjusted pairwise comparisons of EPDS mean ranks between intervention and non-intervention groups. *p < .05, **p < .01

According to the Kruskal–Wallis test, there were significant differences in the EPDS mean ranks in all four measuring occasions between women who started the intervention during different trimesters, or did not attend at all (all *p* < .001). The pairwise comparisons are presented in Fig. 1.

### Perinatal Complications and EPDS Scores

The Mann–Whitney U tests with Bonferroni Corrections showed that EPDS mean ranks were associated with protracted cervical dilation, protracted descent, preeclampsia, IUGR and low birthweight (Table 1), but not with premature birth and GDM (all *p* > .117).

**Table 1:** EPDS mean ranks are connected to some of the perinatal complications

	EPDS mean ranks			
	1st trimester	2nd trimester	3rd trimester	Postpartum phase
Protracted cervical dilation (*N* = 129)	833.07	834.74	825.2	806.54
No complication (*N* = 1523)	825.94	825.8	841.79	1062.14
*p*	.870	.835	.693	.000*
Protracted descent (*N* = 48)	856.44	913.44	1044.55	1047.61
No complication (*N* = 1523)	783.78	781.98	777.85	777.75
*p*	.271	.043^**t**^	.000*	.000*
Preeclampsia (*N* = 52)	817.08	837.52	698.97	1004.47
No complication (*N* = 1523)	787.01	786.31	791.04	780.61
*p*	.636	.414	.135	.000*
IUGR (*N* = 27)	797.91	972.20	942.65	846.83
No complication (*N* = 1523)	775.10	772.01	772.54	774.24
*p*	.791	.018^t^	.042^t^	.366
Low birthweight (*N* = 94)	861.01	972.78	900.84	891.33
No complication (*N* = 1523)	805.79	798.89	803.33	803.92
*p*	.262	.000*	.040^t^	.057^t^

The relationship is significant if *p* ≤ .007 (Bonferroni correction)

*Significant

^t^Tendency relationship

### Perinatal Complications and Intervention

According to the Pearson’s *χ*^2^ tests, no difference was found between the intervention and non-intervention groups in the following perinatal complications: preeclampsia, GDM, IUGR, premature birth, low birthweight, protracted cervical dilation and protracted descent (all *p* > .150).

### Mode-of-birth and EPDS scores

The Mann–Whitney *U* test showed a connection between the EPDS mean ranks and mode-of-birth: those women who had a cesarean section (*N* = 826) had significantly higher mean ranks (1060.92 points) in the postpartum period compared to women who had a vaginal birth (993 points, *N* = 1214), *p* = .007. No significant connection was found during pregnancy, all *p* > .184.

### Mode-of-birth and intervention

According to the Pearson’s *χ*^2^ tests, there was a relationship between intervention and mode-of-birth: women who began the intervention during their pregnancies were significantly less likely to have a cesarean section compared to women who did not participate in the intervention (30.9% vs. 40.69%), *χ*^2^(1) = 4.137, *p* = .042 (Table 2).

**Table 2:** The association between intervention during pregnancy and mode of birth

	Non-intervention group during pregnancy *N*	Intervention group during pregnancy *N*	*p*
Vaginal birth	1121 (59.31%)	76 (69.09%)	.042*
Cesarean section	769 (40.69%)	34 (30.91%)	
Total	1890 (100%)	110 (100%)	

*p < .05

## Discussion

Our results showed significant and tendency relationships between antenatal EPDS mean ranks and obstetric outcomes, such as protracted cervical dilation, protracted descent, IUGR, low birthweight, preeclampsia and depression scores. Not all complication groups showed higher depression scores compared to the control group; thus, our first hypothesis was partly supported. These outcomes confirm previous results (Marcus & Flynn, 2008; Grote et al., 2010; Rahman et al., 2007; Hompoth et al., 2017; Chung et al., 2001), but this study highlighted that with a short, rapid-screening tool, women at risk of these outcomes can be identified and taken care of. Our second hypothesis was also partly supported, as women had higher EPDS scores after cesarean section compared to women who had a vaginal delivery, but we found no significant difference between these groups during pregnancy. In addition to significant relationships, the tendency associations are important to mention, as well, because they also indicate which connections between EPDS mean ranks and outcomes should be addressed through further investigations.

In the literature, about 6.5% of the identified depressed women asked for professional help (Goodman, 2009), but in our sample this figure was 23.5%. The reason for this difference might come from the usually good mother and perinatal nurse connection, in Hungary. Women usually have a good relationship with their perinatal nurses; they trust them, and in the screening program these perinatal nurses suggested women exhibiting possible depression symptoms to seek professional help. However, it should be noted that still less than one quarter of women with high EPDS scores contacted the psychologist. This might be (as women in the intervention group often mentioned) because, in Hungary, there is still a stigma against seeking psychotherapy: most people are still afraid that others would think they are mentally challenged if they ask for psychological help. It is not helpful, either, that extreme or violent psychotic symptoms and behavior are associated with postpartum depression in the media, which makes women confuse the two conditions; consequently, they refuse to ask for help as they do not want to be associated with such aberrant behavior. Public education explaining the differences between the two conditions might help to reverse this negative tendency (Beck, 2001).

Our results showed that the EPDS mean ranks were higher in the intervention groups compared to the non-intervention group. This may be due to the phenomenon that most women in the non-intervention group (80.55%) did not show such pathological depression symptoms during this period, which implies they were better at coping with the situation and its difficulties. It seems, as their pregnancy progressed, they could adapt to the changes and, perhaps, their insecurity decreased. In almost all intervention groups, the EPDS ranks were highest in the trimester when women started the intervention. This might imply that all the women could cope with some of their problems along the way, but reached a point where they felt overwhelmed, and then asked for help. This, too, looks promising, because not every problem needs professional support, as we saw in the non-intervention group, but when there was a need, some women could and did reach out for help. After women attended the interventions, their EPDS ranks tended to decrease in all the subsequent measuring occasions. In addition, the after-intervention EPDS scores were significantly lower compared to the before-intervention scores, which also supports our third hypothesis. According to Milgrom, Schembri, Ericksen, Ross and Gemmill (2011), those women who attended antenatal psychological interventions had lower depression scores in the postpartum phase compared to women who did not attend. In our sample this was not observable, although the mean EPDS ranks of those groups starting the intervention in the first or second trimester did not differ significantly from the EPDS ranks of the non-intervention group. This implies that antenatal psychological interventions started sooner rather than later could help in the prevention of postpartum depression.

Our results showed that women in the intervention group were less likely to give birth to their baby by cesarean section compared to the non-intervention group; thus, our fifth hypothesis was supported. According to Laursen, Johansen and Hedegaard (2009), women with fear of the actual delivery were more likely to have a protracted labor and an emergency cesarean section. During the interventions, some women talked about their fear of childbirth, which became the focus for some of the later consultations. It seems possible that psychoeducational consultations might lead to less-stressful deliveries with fewer cesarean sections, but further investigation is needed to determine the consultations’ contribution.

No significant differences were found in other obstetric outcomes, such as IUGR and low birthweight, between the intervention and non-intervention groups; thus, our fourth hypothesis was not supported. However, this result might be promising, because EPDS ranks were higher in all the intervention groups (in some cases significantly higher) compared to the non-intervention group and, as we presented earlier, some of the complications were associated with higher EPDS ranks. The fact that the statistical analysis revealed no associations with the complications (although those women had higher EPDS ranks) might mean that psychological intervention can provide a kind of protective factor against these negative outcomes, but to confirm this hypothesis further studies are needed.

## Limitations

Patient mobility limited the study, since not all women filled-out the EPDS all four times since, sometimes, women moved away or had only moved to Szeged after the first trimester. Sometimes, perinatal nurses forgot to give the questionnaire to the mothers, as well. Another limitation was the sample size: we believe that the overall sample was big enough; however, the sample sizes of the adverse obstetric outcome groups were quite low. Higher intervention participation would, most probably, be more appropriate and make our results more reliable.

## Conclusions

In conclusion, our results support previous findings that depression symptoms may contribute to adverse pregnancy and obstetric outcomes. However, this effect can be reduced with psychological intervention; therefore, it is important to identify and to treat those women who are at risk of developing depression symptoms. Our research shows that the EPDS screening tool might play an important role in this identification of need.
